# A case for intranasal bumetanide for the management of heart failure

**DOI:** 10.1093/eschf/xvag157

**Published:** 2026-06-01

**Authors:** Alexandria Danyluk, Todd McVeigh, Ivan Nenadic, Joshua Rushakoff, Mark Kittipibul, Marat Fudim

**Affiliations:** Department of Cardiology, Duke University Medical Center, 2301 Erwin Rd., Durham, NC 27701 USA; Department of Cardiology, Duke University Medical Center, 2301 Erwin Rd., Durham, NC 27701 USA; Department of Cardiology, Duke University Medical Center, 2301 Erwin Rd., Durham, NC 27701 USA; Department of Cardiology, Duke University Medical Center, 2301 Erwin Rd., Durham, NC 27701 USA; Department of Cardiology, Duke University Medical Center, 2301 Erwin Rd., Durham, NC 27701 USA; Department of Cardiology, Duke University Medical Center, 2301 Erwin Rd., Durham, NC 27701 USA

**Keywords:** Intranasal bumetanide, Diuresis, Remote cardiac monitoring, Chronic heart failure

## Introduction

In patients with worsening heart failure (HF), conditions such as diuretic resistance or gastrointestinal oedema frequently impair the absorption and efficacy of standard oral diuretics. Because current options are limited when these oral therapies fail, patients traditionally face a gap in outpatient care that often necessitates hospitalization for intravenous (IV) therapy. Bumetanide nasal spray (BNS) has emerged as a novel, non-invasive option that may be particularly beneficial for these patients. Approved in September 2025, BNS is a self-administered, ready-to-use, single-dose nasal spray, similar to Narcan. The medication itself is an intranasal loop diuretic designed for transmucosal absorption to manage fluid overload associated with congestive HF, as well as hepatic and renal diseases. In an open-label randomized controlled trial of healthy subjects, BNS demonstrated more rapid absorption compared to oral bumetanide; while the IV formulation yielded a higher peak serum concentration, BNS showed no significant differences in cumulative urine output, sodium excretion, or safety profile.^1^ Although this formulation holds significant promise for advancing outpatient HF management, further real-world evidence is required to fully characterize its clinical efficacy and safety in patients with worsening HF.^2^ To that end, we present a proof-of-concept case that integrates remote volume management with diuresis achieved with a trial of BNS in an outpatient presenting with worsening HF, illustrating how transmucosal delivery might be utilized to navigate suspected oral diuretic resistance and underscoring its potential as a convenient alternative to intravenous therapy.

## Case summary

A 51-year-old female with a past medical history of HF with preserved ejection fraction (>55%), essential hypertension, insulin-dependent type 2 diabetes mellitus complicated by neuropathy, nephropathy, and retinopathy, chronic kidney disease stage IIIa, obesity class III, and severe obstructive sleep apnoea presented for a routine HF follow-up visit. The patient was initially diagnosed with diastolic HF via heart catheterization at age 45, a phenotype driven primarily by her severe comorbidities, including Class III obesity and Stage IIIa chronic kidney disease. Due to historically challenging volume management, she had previously undergone CardioMEMS placement. Despite remote monitoring and increasing her oral diuretics to a maximum dose of torsemide (100 mg twice daily), she remained persistently volume overloaded with worsening congestion.

At this visit, she reported worsening lower extremity oedema and abdominal bloating. Cardiovascular medications at the time included carvedilol 3.125 mg twice daily, sacubitril/valsartan 97/103 mg twice daily, finerenone 40 mg daily, tirzepatide 2.5 mg weekly, torsemide 100 mg twice daily, hydralazine 50 mg twice daily. She had previously trialled a sodium-glucose co-transporter 2 inhibitor; however, this was discontinued due to recurrent genitourinary fungal infections. Despite remote monitoring and increasing oral diuretics to a maximum dose of torsemide, she remained persistently volume overloaded with worsening congestion. At this juncture, alternative oral therapies such as tolvaptan were deemed impractical due to the requirement for inpatient monitoring during initiation. While the addition of an oral thiazide-like diuretic such as metolazone was strongly considered for sequential nephron blockade, there was suspicion that gastrointestinal oedema might blunt its enteral absorption. Therefore, the decision was made to initially trial BNS as an immediate, as-needed rescue agent to bypass the gut entirely. Notably, following this acute management phase, oral metolazone was subsequently added to her regimen in the following weeks for ongoing maintenance. This clinical decision was further facilitated by the patient's highly accessible $4 copay for BNS, making it an easy and practical alternative. She was not a part of a clinical trial.

At home, she self-administered a total one-time dose of 2 mg of BNS. On the day of the intervention, the patient reported adherence to her background regimen; she took her standard morning dose of torsemide, administered the 2 mg BNS rescue dose in the afternoon, and subsequently took her scheduled evening torsemide dose. Her bilateral lower extremity oedema improved the following day, although she did not notice a notable increase in urine output. Notably, on the day following BNS administration, her weight decreased from 362 to 345 lbs; however, these weights were measured on two different scales. Pulmonary artery diastolic pressure (PAD) detected by her CardioMEMs device declined from 27 on the day of BNS administration to 23 the day following BNS administration. Regarding safety and short-term tolerability, the patient did not report any adverse effects following BNS administration, including local nasal discomfort, epistaxis, dizziness, or symptoms of orthostasis. Refer to *Table 1* for the objective data pre- and post-BNS administration.

**Table 1:** Clinical parameters and timeline before and after bumetanide nasal spray administration

	Pre-BNS (Day 0)	Post-BNS (Day 1)
Additional diuretics	Torsemide 100 mg BID	Unchanged
CardioMEMS PAD	27 mmHg	23 mmHg
Weight	362 lbs	345 lbs
BMI	60 kg/m^2^	57 kg/m^2^
Symptoms	Worsening congestion, bilateral lower extremity oedema	Subjective improvement in lower extremity oedema
Urine output	Not quantified	Not quantified
Blood pressure	127/64 mmHg	115/65 mmHg
Creatinine/eGFR	1.8 mg/dL/35 mL/min/1.73 m^2^	1.9 mg/dL/32 mL/min/1.73 m^2^
Sodium/potassium	140 mEq/L/4.4 mEq/L	139 mEq/L/4.6 mEq/L
Pro-BNP	44 pg/mL	40 pg/mL

BNS, bumetanide nasal spray; BID, twice daily; PAD, pulmonary artery diastolic pressure; BP, blood pressure; BMI, body mass index; eGFR, estimated glomerular filtration rate.

Following the initial administration with BNS, the patient's subsequent 8-week clinical course highlighted the ongoing challenges of managing severe, end-stage HFpEF. Approximately two weeks post-intervention, she developed acute kidney injury, with her creatinine rising from a baseline of 1.6–2.0 mg/dL up to 4.0 mg/dL (BUN 112 mg/dL). This was discovered to be secondary to the unguided self-administration of 2–3 doses of oral metolazone at home. She was instructed to strictly hold all as-needed diuretics, and her renal function appropriately began to recover.

Over the subsequent weeks, however, her venous congestion gradually recurred. By ∼6 weeks after her initial BNS dose, her CardioMEMS PAD pressure had steadily crept back up to 30 mmHg. Although her renal function had returned to baseline (creatinine 1.6 mg/dL, BUN 28 mg/dL), this progressive volume overload ultimately necessitated a hospital admission for inpatient intravenous diuresis. She was successfully diuresed and discharged on with a modestly improved PAD of 26 mmHg and an expected transient worsening of renal function in the setting of diuresis (creatinine 2.0 mg/dL, BUN 62 mg/dL). Notably, her immediate post-discharge course was again complicated by the unguided use of as-needed metolazone, resulting in a recurrent creatinine elevation to 2.8 mg/dL. Ultimately, while the initial transmucosal BNS administration theoretically avoided an immediate crisis and hospitalization by 6 weeks, this follow-up underscores the highly volatile nature of outpatient volume management in patients with complex behavioural barriers.

## Discussion

Current ACC/AHA/HFSA guidelines establish oral loop diuretics as the cornerstone of outpatient HF management. When initial therapies prove insufficient, real-world clinical care involves a nuanced continuum of strategies, including dose escalation, switching to agents with higher bioavailability, and adding adjunctive oral therapies for sequential nephron blockade. However, when progressive venous congestion and suspected gastrointestinal oedema begin to limit the enteral absorption of even robust oral regimens, patients often reach a threshold that traditionally necessitates IV therapy via emergency departments or infusion clinics.^3^ Exploring therapies like BNS highlights a practical niche within this treatment continuum. Transmucosal delivery provides clinicians with an additional, non-invasive tool specifically designed to bypass the congested gut. This case suggests that transmucosal delivery could represent a proactive, patient-centred evolution in care that offers clinicians a potential non-invasive tool to abort acute decompensation episodes early, with the goal to offload burdened healthcare resources and improve treatment access, particularly in rural or lower resourced settings.

**Figure 1:**
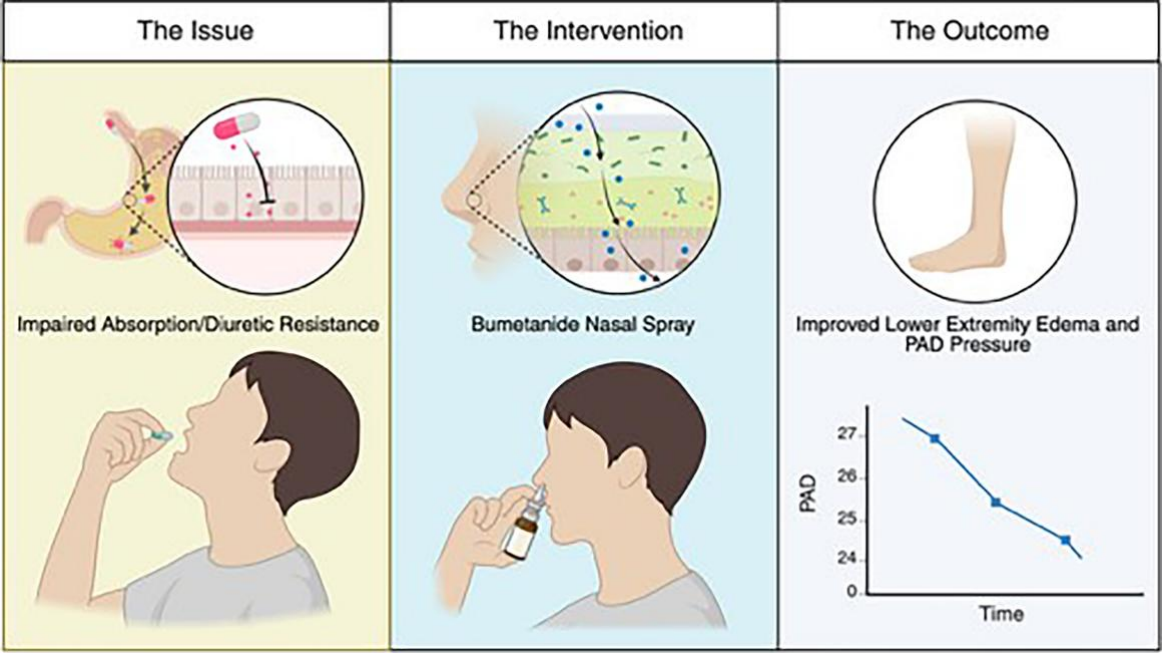
Case summary: intranasal bumetanide as a non-invasive theoretical bridge between oral and IV diuresis

The clinical course of this patient illustrates the potential value of pairing such novel therapeutics with remote monitoring to overcome the physiological hurdles of outpatient volume management (*Figure 1*). As venous congestion escalates in decompensated HF, patients frequently develop splanchnic and gastrointestinal mucosal oedema. We hypothesized that our patient’s reported abdominal bloating was a clinical manifestation of this ‘gut edema’, potentially blunting the enteral absorption of standard oral regimens and contributing to her diuretic resistance.^4^ By utilizing the highly vascularized nasal mucosa, BNS theoretically bypassed this suspected gastrointestinal congestion to deliver effective systemic therapy without the need for venous access. It is important to note that while splanchnic venous congestion leads to profound gastrointestinal mucosal oedema and malabsorption, a theoretical concern exists that elevated central venous pressures could similarly induce nasal mucosal oedema, potentially impairing the transmucosal absorption of BNS. Indeed, product labelling cautions that acute rhinitis or local congestion may alter drug absorption. However, the pathophysiology of systemic volume overload disproportionately affects the gravity-dependent splanchnic vascular bed compared with the venous drainage of the nasal cavity. The objective clinical response seen in this congested patient suggests that, unlike the gastrointestinal tract, the nasal mucosa may remain highly permeable to therapeutic drug absorption even in states of severe systemic right-sided pressures. Nevertheless, clinicians should be mindful of concurrent upper respiratory conditions that could compromise the efficacy of intranasal delivery.

A particularly compelling aspect of this case is the relationship between the patient's subjective experience and her objective haemodynamic response. Although she did not perceive a noticeable increase in urine output—a subjective failure that might have prematurely prompted a hospital admission for IV diuresis in a standard setting—her CardioMEMS device provided objective, real-time data indicating favourable decongestion. The sequential decline in her pulmonary artery diastolic pressures from 27 to 23 mmHg objectively supported the suspected efficacy of the BNS in this instance. A critical limitation when interpreting this objective haemodynamic response is the absence of strictly standardized data regarding the patient's daily dietary sodium and fluid balance. Because this case was managed in a real-world outpatient setting, we cannot completely exclude the possibility that transient, unrecorded reductions in salt or fluid intake contributed to the observed decline in pulmonary artery diastolic pressures, independent of the BNS administration. While her concurrent medication regimen remained unchanged, future prospective studies with tightly controlled dietary parameters are required to isolate the exact haemodynamic impact of transmucosal bumetanide. Furthermore, because this patient's acute volume management was coordinated remotely, our assessment is inherently limited by the absence of serial in-person physical examinations and formal laboratory assessments of natriuresis, and weight tracking was limited to weekly intervals. This proof-of-concept application is especially notable given the patient’s complex comorbidity profile, including Stage IIIa chronic kidney disease and severe obesity, as these conditions frequently complicate volume management and alter pharmacokinetic profiles. Ultimately, this case highlights a promising theoretical role for non-invasive transmucosal drug delivery, suggesting that further study may validate its use in helping clinicians to safely manage complex, diuretic resistant HF at home.

## Conclusions

This proof-of-concept case highlights the theoretical clinical utility of BNS as a non-invasive alternative to intravenous therapy for outpatient volume management. By bypassing gastrointestinal oedema, BNS achieved favourable haemodynamic trends and weight reduction, suggesting diuresis, in a highly comorbid patient with diuretic resistance. Furthermore, coupling this transmucosal therapy with CardioMEMS remote monitoring provided real-time data of improved haemodynamics, preventing premature hospitalization even when subjective increases in urine output were absent. Ultimately, integrating BNS into outpatient care algorithms offers a promising practical patient-centred strategy to manage complex HF at home, with the potential for offloading burdened healthcare resources.

## Take-home messages

- BNS is a promising, non-invasive tool for managing worsening outpatient heart failure, particularly when suspected gastrointestinal oedema compromises the absorption of traditional oral diuretics.
- Further large-scale, real-world studies are warranted to solidify BNS’s role in standard heart failure treatment protocols.

## Declarations

### Data Availability

No data were generated or analysed for this manuscript.
